# *Sensory fields: the visual and the bodily*

**DOI:** 10.1007/s11098-022-01838-x

**Published:** 2022-06-09

**Authors:** Carlota Serrahima

**Affiliations:** Universitat de Barcelona, Barcelona, Spain

**Keywords:** Sensory field, Somatosensation, Bodily experience, Bodily ownership

## Abstract

Philosophers of perception have been readier to postulate the existence of a visual field than to acknowledge sensory fields in other modalities. In this paper, I argue that the set of phenomenal features that philosophers have relied on when positing a visual field aptly characterise, mutatis mutandis, bodily sensation. I argue, in particular, that in localised bodily sensations we experience the body as a sensory field. I first motivate this claim for the case of haptic touch, and then generalise it to other kinds of bodily sensation. I demonstrate the theoretical fruitfulness of this notion of a bodily field for the debate on the phenomenology of bodily ownership.

## Introduction

In analytic philosophy of perception, there has traditionally been much more readiness to recognise the existence of a visual field than there has been to acknowledge sensory fields in other modalities. Touch, in particular, has been said to lack a sensory field (Strawson, 1959; O’Shaughnessy, 1989, 2008; Martin, 1992, 1993, 1995).

In philosophical discussion of visual perception, the notion of sensory field has been invoked to explain the spatial nature of visual experiences: in vision, objects are ordinarily experienced as contained, and distributed within, a space that is itself also perceived, and which demarcates the area within which the seen objects are located – i.e. the sensory (in this case, the visual) field. The lack of a sensory field in touch has often been defended by comparing visual and tactile perception and highlighting the differences between the two as to how space is experienced. In an often quoted passage, Strawson wrote that “[e]vidently the visual field is necessarily extended at any moment, and its parts must exhibit spatial relations to each other. The case of touch is less obvious: it is not, e.g., clear what one would mean by a ‘tactual field’” (1959, 65). O’Shaughnessy (1989, 2008) went into detail about the spatial disanalogies between vision and touch that suggest that touch “involves the use of no mediating field of sensation” (1989, 38). According to O’Shaughnessy, a central difference between sight and touch is that tactile sensations occur in three-dimensional space only if they are sensations at, and of, a part of a *particular body* in space; whereas visual sensations are presented in the visual field independently of “landing upon” material objects (2008, 217).

Martin (1992, 1993, 1995) took up this idea. He did not go so far as to say that touch involves no sensory field at all, but he did claim that the role the visual field plays in vision is not played by anything in the tactile domain (1992, 197). His reasoning partly recapitulates O’Shaughnessy’s: the visual field is not fully occupied by seen objects, for we perceive empty space as well. In this sense, “the space within which the objects are [visually] experienced as located is itself part of, or the form of, the experience” (ibid., 198). In contrast, because tactile perception depends on bodily awareness – that is, on the location of sensations in the body (Richardson, 2011) –, tactile sensations present space only insofar as it is occupied by the body. Instead of objects located *within* space, Martin says, what one experiences in touch are parts of a single body and happenings within it. We have no tactile awareness of empty space because tactually felt space is primarily bodily space (1993, 215).^1^

Contesting this tradition of scepticism about sensory fields beyond the visual, some fairly recent empirical studies on touch have been presented as speaking in favor of a notion of tactile field. According to these studies, it makes sense to talk about “skin space”: hosting a network of receptive fields, the skin actually supports the representation of continuous spatial patterns that integrate various individual tactile stimuli, hence representing, not only the locations of the actual stimuli applied on the skin, but also the relations between these individual locations (Haggard & Giovagnoli, 2011; Haggard et al., 2017; Fardo et al., 2018; Cheng, 2019). On these grounds, it is arguable that touch is similar to sight in that it represents space beyond the specific locations of its individual objects. To some extent, this brings us closer to being able to claim outright that there is a sensory field in touch. Yet, it is still the case that, as the philosophers mentioned above pointed out, tactile space beyond individual objects of touch is not quite *empty* space: it is skin space, and hence occupied by the body.^2^

This paper contributes to extending the notion of sensory field to the tactile domain. Methodologically, however, it does so on the grounds of phenomenological considerations: in other words, it does so on the grounds of considerations similar in kind to those that have actually led philosophers to scepticism about non-visual sensory fields. On the grounds of phenomenology, I will argue, we have principled reason to talk about a sensory field in the tactile modality. And not only this: the same kind of reasons invite talking about other bodily sensations – in particular, localised bodily sensations – as involving a sensory field as well.

Bodily sensations, or somatosensory states, are states of bodily awareness.^3^ Speaking generally, all bodily sensations differ from vision in the sense observed by O’Shaughnessy and Martin. While vision conveys a number of objects distributed within space, the body always monopolises bodily sensation, in the sense that “it seems to one that one is aware of a region of space within which [sensations] are located only in so far as one is aware of some *thing* occupying that region of space. Hence the fact that one does not feel these sensations to be in a space but not in a body” (Soteriou, 2013, 120). This can certainly be used as ammunition by the sceptics about sensory fields: outside of visual perception, not only touch, but the whole somatosensory domain, is devoid from the kind of spatial phenomenology indicative of the involvement of a sensory field.

In this paper I do not intend to deny the distinctive status of the body in tactile perception or somatosensation more generally. Quite the contrary, my strategy is to take a close look into the phenomenology of bodily awareness in these modalities in order to argue for the following claim: in tactile perception, and in localised bodily sensations more generally, *the body itself* is experienced as a sensory field.

What does it mean, however, to experience the body *as a sensory field*? In my approach to this issue, I will also rely on a comparison between visual perception and somatosensation. Despite the numerous differences that, it has been acknowledged, exist between the phenomenologies of the two modalities,^4^ there are also some substantive similarities between them. I will argue that the set of phenomenal features that philosophers have commonly focused on as main motivation to posit a visual field characterise, mutatis mutandis, bodily experience in somatosensation. Hence, if we have reasons to think of vision as involving a sensory field, then we also have reasons to think of somatosensation in this way. My claim that, in somatosensation, we experience the body *as a sensory field*, means that we experience the body in somatosensation as philosophers of perception have argued we experience the visual field in visual experiences. Crucially, however, the phenomenological features that bring this to light do not concern exclusively our experience of space in each modality. Rather, they concern another feature central to sensory fields: the relation they bear, and are experienced to bear, to external objects and their properties.

The structure of the paper is as follows. In Sect. 2, I zoom in on the notion of visual field by focusing on Gibson’s and Peacocke’s arguments for its existence. Out of this discussion, I extract four main phenomenological features, putatively present in visual experiences, which these theorists take to warrant postulation of the visual field. On the grounds of these four features, I offer a definition of sensory field. In Sect. 3, I show that the very same four features characterise bodily experience in haptic touch. This gives me a principled reason to claim that, at least in haptic touch, the body is experienced as a sensory field. What is more: precisely because it is the body that plays the role of the sensory field in touch, those four features might apply more clearly, and less controversially, to the haptic realm than they do to the visual. After considering an objection to my characterisation of haptic touch in Sect. 4, in Sect. 5 I propose an extension of the foregoing argument about haptic touch to localised bodily sensations in general. I will call the sensory field involved in bodily sensations *bodily field*.

Localised bodily sensations range from, say, raising an arm and proprioceptively feeling it raise, to nociceptively feeling a sharp pain in the toe, through feeling an itch on the thigh, a cold ice cube on the forehead, or plain tactile sensations. To be clear, subsuming such a wide variety of somatosensory states under a general notion of sensory field, i.e. the bodily field, is not uncontroversial. In their defence of a tactile field, for instance, Haggard et al., (2017, 110) point out that other somatosensory states “have quite different spatial properties, and may lack a field-like organizing principle”, and they suggest that “[h]aving a sensory field or not might … thus provide a logical starting point for modality individuation” within somatosensation.

There are several ways of individuating sensory modalities, some of which most illuminating when engaging with certain explanatory projects; some others when engaging with others. I do not intend to make a strong claim about sensory individuation in this paper. More limitedly, however, I do want to stress what I take to be an important explanatory virtue of considering at least some somatosensory states under the light of a notion of sensory field – a phenomenologically based notion, extracted from commonplace assumptions in the philosophical literature on visual fields. I will conclude the paper (Sect. 6) by highlighting this virtue. As I will point out, the idea that in somatosensation we experience the body as a sensory field can shed light on what, it has been acknowledged, might be a phenomenal commonality accross somatosensory states: that we have a sense of ownership for the body that we experience in this way (Chadha, 2018; Vignemont 2018; Bradley, 2021; Bermúdez, 2020).^5^

## Sensory fields

Defenders of the notion of a visual field have often relied on the following phenomenological datum about visual experiences: some properties relevant to the specification of the experience’s what it is likeness are not possessed by the experiences in virtue of their representational content (Peacocke, 2008).^6^ For an illustration of this datum, consider the following descriptions of different visual experiences:

[Bullet train]: Suppose you are in a flat area of Japan. You may perceive a bullet train in the far distance, and you may perceive it as moving extremely fast. Your experience has the representational content that it is moving fast. But it may be moving rather slowly across your visual field, and that too is something of which you can become aware. It may be moving across your visual field at the same rate as a nearby cat that is moving very slowly across a path in front of you. (ibid., 2008, 15–16)

[Railroad tracks]: [E]quidistant edges of man-made structures appear to get closer together … as they recede in the distance. When looking down a highway or railroad tracks the effect is strong … [H]owever, two observers may differ in describing what they see. One will report that the parallel lines definitely converge as they go off toward the horizon; another will insist that the rails do not converge since they are visibly equidistant. Each scene is perfectly clear to each observer, but they are contradictory to each other … [This fact] suggests only that there may be two kinds of seeing. (Gibson, 1950, 35)

In [Bullet train], your visual experience involves various properties. One of them, which we may call Rapidity, is attributed to the train in the experience: your experience represents an extremely fast train. Another one, arguably, is Slowness. Slowness is an experiential property, in the sense that it informs the phenomenology of your experience. You can switch attention and focus on Slowness: you can become aware of how the train moves, from one extreme to the other of the section of trail that you see, as slowly as a slow cat near you. This switch of attention makes Slowness phenomenally salient in the experience. In any case, however, Slowness is *not* attributed to the train in the experience: your experience does not represent a slow train.

[Railroad tracks] can be similarly described. The visual experience in [Railroad tracks] represents parallel tracks. Hence, when looking down a railroad track you may simply see how the tracks run parallel to one another, as the second observer does. In this sense, your experience involves a property that we may call Parallelism. But it is a known fact that, in some other important sense, parallel lines are seen to converge as they advance towards the horizon. This is what the first observer in Gibson’s example reports. Thus, the experience also involves Convergence. Convergence certainly informs the phenomenology of the experience. Parallelism and Convergence can both be made phenomenally salient by switching attention: the two “kinds of seeing” that Gibson talks about pick out these two possible focuses. However, even when Convergence is phenomenally salient, it is not attributed to the railroad tracks in the experience: your experience does not represent convergent tracks.

These examples aim to show that the phenomenology of at least some of our visual experiences is such that, for some of the properties involved in the experiences, they are not properties assigned to the represented objects in the experiences. Both Gibson and Peacocke defend that, for all visual experiences, we can perform switches of attention so that properties like Slowness in [Bullet train], or Convergence in [Railroad tracks], become salient in the experience (Gibson, 1950, 26 − 7; Peacocke 2008, 8).

At this point, then, a question emerges: what are Slowness and Convergence assigned to in the experiences? These theorists defend that these properties “need not be instantiated by the experience itself in the head of the experiencer” (Pautz, 2010, 259). Rather, Slowness, Convergence, and the like, motivate positing a further relatum to visual experiences: the visual field. The visual field is, precisely, the bearer of those experiential properties that the objects of visual experience are not represented as having.^7^ And more importantly: Slowness, Convergence, and the like are given in the experience *as properties of (regions of) the visual field*, insofar as they are not presented as properties of objects. When we focus on Slowness or Convergence in the experience, we are aware of the visual field as such.

It is important to note that, when the visual field and its properties are phenomenally salient as such, the experience is not fully transparent with respect to the objects represented. Harman famously predicted that, if you look at a given object, “the only features there to turn your attention to will be features of the presented [object]” (1990, 667). In this sense, Harman said, your visual experience of the object will be transparent. Our ordinary experience as of the bullet train in the far distance will indeed be, by default, a straightforward experience as of a fast train. But let us concede, for the sake of argument, that we can turn our attention to Slowness in [Bullet train]. While focusing on Slowness, this property undermines the transparency of the experience as of a fast train, for it is clearly not, and it is clearly not presented as, a feature of the train. Otherwise put, Slowness *veils* the fast train, and Convergence veils the parallel tracks, when we focus on these properties in the experiences.

One last central feature of the visual field as presented by its defendants is its canvas-like function. On the one hand, the visual field has boundaries, and we are aware of these boundaries in visual perception. Gibson, for example, writes that “[i]f you keep your eyes fixed but put your attention on the periphery of the field (a trick that may require practice) you can observe that things are visible only to a limited angle to the right and left and to an even more limited angle upwards and onwards. These boundaries, it is true, are not sharp … and they are hard to notice … but they are nevertheless present” (1950, 27). On the other hand, the boundaries of the visual field are the limits within which the seen objects are presented. Hence, crucially, for experiential properties to be salient as properties of (regions of) the visual field is for them to be salient as properties of the area within which the seen objects are presented.

Based on the features of the visual field just discussed, discovered upon reflection on the examples, a notion of *sensory field* that encompasses all such features can be defined. For a given sensory experience type, the sensory field.

i) is the bearer of those properties involved in experiences of the type that are not assigned to the objects represented in the experiences;^8^
ii) has perceived boundaries, and these boundaries demarcate the area within which the perceived objects are presented;
iii) because its properties inform the phenomenology of the experiences, the subject can focus on them, making the field and its properties phenomenally salient as such;^9^ and
iv) when this is the case, the experience becomes non-transparent with respect to the objects represented in it.

Clauses i) to iv) summarise the phenomenal features of visual perception that theorists have commonly organised into the notion of a visual field.^10^ In the sections to follow I will defend that the same phenomenal features characterise, mutatis mutandis, bodily experience in somatosensation. Regardless of how accurate the analysis of visual perception in terms of a visual field is, the fact that the phenomenological intuitions behind this analysis extend to somatosensation gives principled reason to talk about somatosensation in terms of a sensory field. My aim in the upcoming sections is indeed to argue for the claim that in somatosensation we experience the body as a sensory field. As mentioned above, in order to make my case I will first motivate this claim for haptic touch. Then I will proceed to arguing that the features of the phenomenology of haptic touch that support the claim that it involves a sensory field do not show up only in touch, but in all localised bodily sensations.

## A sensory field in touch

Haptic touch is the perception of objects by actively exploring them with a body part. Feeling the relief of the neck of my water bottle by caressing it with the tip of the fingers, for instance, is a case of haptic touch. Haptic touch is a type of exteroceptive perception: it is mainly object-directed, in the sense that one engages in it in order to perceive the properties of objects other than the body. For example, I might want to count the precise number of rings of the neck of the bottle by touching it. In this sense, the external objects perceived in touch plausibly are the objects represented in tactile experience.

However, when touching the neck of the water bottle I do not only feel the wavy metal, but I also feel the pressure that it exerts on my fingers. In fact, haptic experiences are typically said to be dual: they involve awareness both of external objects and of the body (Richardson, 2011; Matthen, 2021). The involvement of bodily awareness in haptic experiences is crucial to the point I want to make here. Tactile experiences have external objects only insofar as a region of the bodily surface is felt to be in contact with what is touched. With this in mind, consider again the water bottle example as restated below:

[Water bottle]: I caress the neck of my water bottle with the tip of my right hand fingers. I tactually feel the relief of the metal. Suddenly, I also feel a bulge on the tip of my index finger. I rub that area of the finger against the metal a bit more insistingly, trying to figure out how big the bulge is.

[Water bottle] affords a description in which the general notion of sensory field defined above applies. The experience described in [Water bottle] involves a property that we could call Wavyness: I tactually perceive the wavy neck of the water bottle. The neck of the water bottle exerts some degree of pressure on my right hand fingers, and in this sense, my tactile experience also involves awareness of some properties of my fingers. That is why I can find out about the irregularities on my fingers’ skin on the grounds of touching the bottle. Let us say, then, that the experience described in [Water bottle] also involves awareness of the Depression of the fingers against the bottle.

Importantly, the felt surface of the fingers provides the area within the limits of which the bottle is presented. Indeed, the external objects of touch are perceived as matching the felt bodily area they are in contact with, where the extension and limits of this perceived area are defined by the contact with objects. Hence, the experience presents Depression as a property of the area within which the bottle is presented. By the same token, Depression is not experienced as a property of the water bottle. Because Depression is an experiential property informing the phenomenology of my tactile experience, I can switch attention and focus on it while touching the bottle.

One fruitful way of cashing out the point about the saliency of either objects’ or bodily properties in touch is by seeing it as a case of a more general perceptual phenomenon, namely the distinction of the proximal and the distal in perception. As Matthen (2021, 200) points out, in general, external stimuli cause, on the one hand, a proximal experience, that corresponds to the state of the sensory receptors when registering the stimulus, and on the other hand, a distal experience revealing the properties of the external object of perception. In the case of touch, our awareness of our bodily condition when in contact with objects – e.g. of the Depression of the fingers – corresponds to the proximal experience, constructed out of the response-levels of cutaneous receptors; whereas our awareness of the touched objects – our perception of e.g. Wavyness – corresponds to the distal experience.^11^ In all perceptual experiences, “[o]ften, the proximal experience is in the background and requires an act of deliberate attention” (ibid.). In particular, in touch “[m]ost observers are primarily aware of the distal features of the things they touch” (ibid.). However, bringing the body to light is possible by deliberate switches of attention.

On the foregoing description of [Water bottle], a sensory field as characterised above appears to be involved in it. Let us provisionally call this field *tactile field*. This description of [Water bottle] immediately brings to light one peculiarity of the tactile field. As a matter of fact, it is relative to *the body* that the external objects of haptic touch are presented. And more importantly: properties such as Depression, which I have appealed to in order to motivate the claim that haptic touch involves a sensory field, are presented to me in the experience as properties of the body.

This already announces the proposal I want to pursue here: in haptic touch, the body itself is experienced as a sensory field.^12^ Let us resort to clauses i) to iv) from the previous section in order to articulate this claim clearly. In haptic touch, *the body* is presented to us as

i) bearing those properties involved in tactile experience that are not assigned to the objects represented in the experience (e.g. Depression);
ii) having boundaries, where these boundaries demarcate the area within which the touched objects are presented;
iii) because these properties (e.g. Depression) inform the phenomenology of the experiences, we can focus on them, making the body and its properties phenomenally salient in the experience; and.
iv) when this is the case, the experience becomes non-transparent with respect to the objects represented in it.

Clauses i) to iv) in the previous section summarised the phenomenal features of visual experiences that, we have seen, motivate positing the participation of a sensory (a visual) field in those experiences. A few comments on the application of the clauses to the haptic case are now in order.

For a start, clause i) is in principle compatible with the following: at least in some sense of “representation”, the body is part of what is represented in haptic experiences, alongside the touched objects. We can get a grasp of what the relevant sense of “representation” is here by thinking, again, of Slowness in [Bullet train]. Slowness is not presented in our visual experience as a property of the seen train. But this is compatible with the claim that it is presented, in the experience, as a propery of a relatum of the experience – instead of as a property of the experience itself. Indeed, the visual field is supposed to occupy the place of this relatum, according to its supporters.

In the same vein, the body can be thought of as part of the content of sensations. In fact, this is less controversial in the bodily case than it is in the visual field case. The visual field is not obviously presented to us as an individual object in visual perception. In contrast, there is a clear sense in which the body is presented to us as an individual object in bodily sensations,^13^ and in touch in particular. Hence, in this respect, there is one task that seems more straightforward for the theoretician of bodily fields than it is for the theoretician of visual fields, namely the identification of the actual, worldly entity the field corresponds to.

At this point, one might worry that the phrasing of clause i) – sensory fields bear those properties involved in the experience that are *not* assigned to the *represented* objects – is mere wordplay then: after all, the sensory field is also part of what the experience represents. If read together with clause ii), though, it is clear that clause i) is not mere wordplay. By positing the existence of a visual field, authors intend to capture a phenomenologically manifest asymmetry in the representational status of the field vis à vis that of objects: the visual field is presented as standing in certain relation with the objects, namely as providing the area within which the objects are represented. Analogously, haptic experiences convey the body, yet as having a special status relative to the touched objects: that is, as providing the area within which the touched objects are represented. In this sense, sensory fields have a structuring role with respect to the objects represented in experience (Richardson, 2009).

Clause iii), in turn, says that bodily properties inform the phenomenology of haptic experiences, and hence that we can focus on them. We thus make the body and its properties phenomenally salient in the experience. Importantly, this equates to saying that the body is salient *as a sensory field* in the experience – insofar as, per i) and ii), it is salient as the area within which the objects are presented. In normal conditions, all haptic experiences involve bodily properties like Depression, even if only recessively. Making these properties salient in the experience just is undermining the transparency of our experience as of a given external object, as clause iv) states. Just like Slowness did to the fast train, or Convergence to the parallel tracks, Depression veils the wavy water bottle.

## An objection

Before extending my argument beyond haptic touch, I shall address an objection, suggested by the claim that the body is part of what is represented in haptic touch alongside the touched objects. In light of this claim, the asymmetry defended between the body and the external objects of touch, by which the former is experienced as a sensory field, might be found ad hoc.

The objection goes like this. Let the haptic experience of touching the water bottle have the body as its represented object. Holding on to this assumption, we can now run the description of the case as follows: Wavyness is a property involved in this haptic experience, not attributed to the object represented in the experience. Rather, Wavyness is attributed to the water bottle. The bottle, in turn, is that against which the body is represented in the experience. Thus, on this alternative description, the touched object constitutes the backdrop against which the body is perceived. What reasons do we have, then, to think that the body, and not the touched object, has the status of a sensory field in the experience?

In reply to this objection, two things can be said. Firstly, we can resort to the observations about transparency made above. In haptic touch, attention is typically placed on non-bodily objects: in order for the body to be phenomenally more salient than the objects touched, an effortful switch of attention is needed. Touched objects such as water bottles do not have the status of sensory fields in touch for the same kind of reasons why trains or railroad tracks do not have this status in vision: exteroceptive experiences, both visual and haptic, are directed towards the external objects. In this sense, those properties of experiences that we make salient by switching attention, and which are not attributed to the external objects, veil the external objects. The body is perceived as a sensory field, and the water bottle isn’t, because it makes sense to say that the former veils the latter, but not quite the other way around.

This first reply to the objection relies on how attention is *typically* distributed. The definition and argument in the foregoing sections are indeed partly based on observations about typical attentional distribution in vision and haptic touch. What this first reply does, then, is to show that the alternative description of the water bottle case presented in the objection is imperfect in this regard, given how the experiences of actual perceivers in normal conditions are.

But one might worry that this reply leaves open a counterfactual scenario to which my proposal seems somewhat vulnerable. For what if, for some perceivers, their attention were primarily drawn to bodily properties in haptic touch, so that only with an effortful switch of attention they were able to focus on the properties of external objects? Would external objects have the status of a sensory field in this scenario, or are there still reasons to think that the body would?^14^ The concern here ultimately is that the asymmetry defended, between the body and the external objects of touch, be *merely* a matter of how our attention happens to be distributed in ordinary perception – so that changes in attentional distribution would suffice for the asymmetry to fail – instead of touching upon something essential about the role of sensory fields in experiences.

This leads to the second set of considerations in reply to the objection. The asymmetry between the body and the external objects of touch is indeed a substantive one – one that, as mentioned above, has to do with their respective representational status: sensory fields have a structuring role with respect to the objects represented in experiences. In principle, a structuring feature of items of a type is something that remains relatively stable across items of the type. In the case of sensory fields, their structuring role in experiences was spelled out, in the definitions above, in terms of how they define the area within which the objects perceived are presented.

In this sense, for experience types that involve a sensory field, the field is a manifestly stable feature across experiences of the type (cf. Skrzypulec, 2022): for instance, in the case of the visual field in visual experiences, the field corresponds to the bounded cone, with its apex where our eyes are, within which objects of vision are possibly seen. In turn, in the case of haptic experiences, the stable feature is the body: while touch can take a wide variety of objects as its represented external objects, these objects are consistently presented against the backdrop of one and the same body. In other words, what remains comparatively constant across haptic experiences is the body, and not external objects. The second reply to the objection is thus this: the external objects of touch are not likely to play the role of a sensory field in haptic experiences because, possibly varying from one experience to another, they do not display the component of stability typical of structuring features of experiences.

## Generalisation

### Sensory fields without objects

In this section, I aim at showing that the features of the phenomenology of haptic touch exploited in support of the claim that touch involves a sensory field show up, mutatis mutandis, in all localised bodily sensations. In other words, I aim at showing that, in localised bodily sensations other than haptic touch, we experience the body as a sensory field.

One initial source of skepticism about the thesis that bodily sensations beyond haptic touch involve a sensory field is that most of these bodily sensations do not represent non-bodily objects. When I raise an arm and proprioceptively feel it raise, my experience as of a raised arm is limited to the arm itself: it does not represent any non-bodily object out there in the world. For another instance, when I nociceptively feel a sharp pain in the toe, my experience conveys the toe: it does not represent worldly objects beyond my foot. The notion of sensory field I have been dealing with here, though, is defined in relation to the external objects represented in sensory experience. How, then, can we make sense of the claim that bodily sensations other than haptic touch involve a sensory field?

The reply to this question lies in the following: we need not think of sensory fields as figuring only in those experiences that *actually* represent objects. Rather, we can think of sensory fields as only *possibly* occupied by objects. Sensory fields thus characterised can still bear experiential properties, which would anyway not be assigned to objects in case the experience represented some.

This refinement to the notion of sensory field is not a bespoke modification of the notion defined in Sect. 2. The notion of visual field, as it has traditionally been cashed out by its defendants, is already thus refined. Consider another example offered by Peacocke in support of the visual field:

[Noonday sun]: When you close your eyes and point your head in the direction of the noonday sun, you have a visual experience in which there are colours and shapes, and usually some motion, in your visual field. It does not thereby look as if there are objects or events in your spatio-temporal environment (2008, 8-9).

Visual experiences like the one described in [Noonday sun] are common. The visual experience in [Noonday sun] does not represent any particular object in the subject’s environment. Yet, there is something it is like to undergo the experience, for one sees some free floating patches of colour. In other words, there are some properties relevant to the phenomenology of the experience, such as color properties. It follows that these experiential properties are not instantiated in virtue of the representational content of the experience (ibid.). The visual field is posited precisely as the bearer of this kind of properties. Hence, it is very centrally posited as figuring in, and explaining the phenomenology of, visual experiences that do not actually represent external objects, where this is patently so for the subject.

It is important to note that external objects *can* anyway be represented within this field, though. According to visual field defendants, if external objects were eventually represented within the field, the properties of the field would not be assigned to these objects.^15^ Suppose that, after pointing your closed eyes to the sun, you open them and let your gaze rest on a surface in front of you. You may then experience an afterimage: you may keep seeing those free floating patches of color, now occluding some bits of the surface. Yet, you will not take these patches to be objects in the environment, nor stains on the surface itself. Color properties are presented to you in these examples *as properties of the visual field*, since they are not assigned to objects represented in the experience – in some cases, as in [Noonday sun], simply because there are none. In any case, the field as such is phenomenally given, apparent to the subject in virtue of a contrast with how external objects either are, or would be, presented in the experience in which the field participates.

Just to illustrate the same point further, consider Gibson, when he writes that afterimages “do not disappear when the eyes are closed”, and adds that “[w]hen the eyes are open, [afterimages] appear to be superposed on the objects of the visual world but not to be objects themselves, and they have a filmy insubstantial look … After-images, therefore, are localized with reference to the visual *field*. Insofar as they have a visible location, it is in this field” (1950, 32). Experiential properties without represented objects are also central to Gibson’s argumentation in support of the visual field. To repeat, we need not actually see anything – our eyes might be closed – in order for the visual field to be salient as such in our visual experience, its defendants claim. Besides, the properties of the field presented to us in these circumstances do not subsequently appear to us as properties of seen objects when our eyes are open.

There is no reason to think that this refinement to the notion of sensory field does not carry over to the tactile field. That is, we need not think of the tactile field as figuring only in those bodily sensations that involve actual tactile intercourse with objects: rather, the tactile field can be thought of as only possibly filled in with objects. As a matter of fact, our body allows us to somatosensorily access external objects *if* it establishes contact with these objects, namely if actual tactile intercourse occurs. As I will argue in the next subsection, there are reasons to think that this fact is built into the phenomenology of bodily awareness in somatosensory states beyond haptic touch. Henceforth, I will resort to the expression *bodily field* to refer to the somatosensory field so conceived.^16^

### The sense of boundedness and tactile expectations: the bodily field

My argument in favour of the involvement of a tactile field in haptic touch relied on a phenomenologically manifest asymmetry in the representational status of the field vis à vis that of objects. This asymmetry was identified by appealing to the possibility of switching attention between experiential properties – a method inherited from discussion of vision and the visual field. Now we are considering somatosensory states for which switches of attention between bodily properties and properties of objects are not available, since no external objects are represented in them. However, I contend, a relevantly similar asymmetry between the body and what lies outside of it is phenomenologically manifest as well in localised bodily sensations, insofar as these sensations involve a *sense of boundedness*.

Recall [Water bottle]. The haptic experience described in [Water bottle] involved Wavyness and Depression. In this situation, we said, our attention would normally be directed to the wavy bottle, but we could still focus on the fingers, depressed against the bottle’s surface. One important feature of [Water bottle] is that Wavyness and Depression are felt to be instantiated at different locations (Matthen, 2021, 200): they are felt to be instantiated at one and the other sides of the perceived boundary of the touching hand. In [Water bottle], the boundary of the hand is phenomenologically salient as in contact with the bottle. Hence, switching attention between Wavyness and Depression consists in shifting attention from beyond the boundary of the hand, to the opposite side of this boundary, namely the bodily side. This saliency of the bodily boundary in the experience is what we call *sense of boundedness*.

Crucially, the sense of boundednesss is not exclusive of haptic touch. It is now accepted that localised bodily sensations involve a sense of boundedness (see e.g. Gallagher 2017; Vignemont, 2018). This idea was introduced in the contemporary discussion by Martin (1992, 1993, 1995), in the context of the discussion of touch and bodily awareness mentioned in the Introduction. The idea is that, by having sensations as of certain bodily events occurring at a given bodily location, we feel that the body is bounded. Bermúdez (2018, 2020) has recently revived this idea in what he calls a Boundedness principle: “bodily events are experienced within the experienced body (a circumscribed body-shaped volume…).”^17^ As he puts it, the principle emphasizes how, in localised bodily sensations, we feel bodily events as in relation “to the *experienced spatial bounds* of the body” (2020, 113. My emphasis). For an illustration, suppose that you raise an arm above your head and proprioceptively feel it stretch (Martin, 1993, 210-2; 1995, 271). In this situation, you feel your arm as having a certain extension: your arm appears to extend to at least the points in space where you are feeling it to be.

In haptic touch, the sense of boundedness came with a duality between the body – e.g. the depressed fingers – and the actual object of touch, which lies outside of it – e.g. the wavy bottle. Just as the sense of boundedness does, this duality between the body and what lies outside of it generalises to all localised bodily sensations. Feeling the body as bounded means feeling it to be disposed within a space that goes beyond that in which we feel it to be. Hence, in localised bodily sensations, “in addition to having some sense of the extent of one’s body, one also has some sense of the world extending beyond [the body’s] limits” (Martin, 1993, 212).

Indeed, the information one gets about anything beyond the bodily boundaries on the grounds of somatosensory states is scarce: actual contact with objects missing, one only gets “some sense” that the world extends beyond the body’s limits. But that does not undermine the role of the body as a sensory field in these states. I contend that, because of the duality described, the sense of boundedness in bodily sensations is eo ipso a sense of *possible* tactile intercourse with objects. The body is experienced as a sensory field in localised bodily sensations because it is experienced as a bounded object that *can* give us somatosensory access to anything beyond its boundaries.

Compelling theoretical support for this idea can be found in Vignemont’s (2021) notion of *tactile expectation*. Vignemont has recently called attention on the specific computational principles that govern *visual* perception of peripersonal space, namely the space immediately surrounding the body. As she writes, “[o]bjects in peripersonal space are not yet in contact with the body, but they may soon be” (176), and this is wired into our cognitive architecture: visual perception of peripersonal space is determined by a mainly predictive function. “The perceptual system”, Vignemont writes, “predicts that the location of objects that are for now only close to the body will soon be on the skin itself ” (177). In this sense, when seeing objects in peripersonal space our perceptual system constantly forms tactile expectations: it *perceives* objects in peripersonal space *as* soon to be in touch with the body. This peculiar encoding of peripersonal space is crucial for survival, for it elicits quick protective behaviour.

Importantly, on Vignemont’s view, not only tactile expectations determine visual processing of objects in peripersonal space, but they also have an impact on the phenomenology of our visual experiences. Visually expecting tactile intercourse with an object involves a phenomenally salient sense that the object may have an effect on us. In other words, we have a (visual) sense of possible tactile intercourse with objects.

Note that, as described by Vignemont, tactile expectations are essentially expectations of the effect of external objects on the body. Hence, the very idea of a tactile expectation rests on the assumption of a complementary claim about how we are aware *of the body* as soon to be affected by objects. Indeed, what counts as peripersonal space at a given moment depends on the location of the bodily boundaries at that moment. As we move through space, some objects actually enter, and some others actually leave, the region of space in which tactile interaction is immediately possible, in virtue of the current whereabouts of our bodily boundaries. Hence, representing space as peripersonal in visual perception at a given moment partly depends on the representation of the location of the bodily boundaries at that moment. And arguably, the representation of the location of the bodily boundaries at a given moment is very importantly contributed by the sense of boundedness – namely, by where somatosensory states indicate these boundaries to be.

Tactile expectations shed new light on the sense of boundedness. Our sense of the bodily boundaries plausibly is the complementary phenomenon, in somatosensation, to tactile expectations in peripersonal vision: it is a sense of where tactile intercourse with external objects could occur; a sense that the body may (soon) be affected by anything lying beyond it.

Let us now resort to the four clauses used above as indicators of the involvement of a sensory field in a given sensory experience type. Relying on the considerations about localised bodily sensations in this section, we can now make explicit how the clauses generalise, mutatis mutandis, to these sensations. In localised bodily sensations, *the body* is presented to us as^18^

i) having boundaries, where these boundaries demarcate the area within which external objects would be presented were they to get in touch with the body;
ii) bearing properties not assigned in the sensation to anything beyond the bodily boundaries (i.e. bodily properties). Per i), these properties are assigned, in the sensation, to the area within which external objects would be presented if they got in touch with the body;
iii) because these properties inform the phenomenology of the sensations, we can, and often do, focus on them in the sensation. In other words, the body and its properties are phenomenally salient in somatosensation; and
iv) because the sensations are just about bodily properties, they are opaque relative to external objects beyond the body.

Even if, as iv) indicates, the only specific object conveyed by the sensations is the body itself; and even if, therefore, we might focus only on the body when we undergo certain sensations, such as that of raising an arm or that of a sharp pain in the toe; the body is experienced as a sensory field in bodily sensations insofar as it is experienced to stand in a certain relation with the external world: it is experienced as that which can give us somatosensory access to it. The asymmetry in representational status between the body and what lies beyond it is preserved, and more importantly, is phenomenally salient, in localised bodily sensations.

These considerations conclude my defence that, in localised bodily sensations, we experience the body as a sensory field. In other words, they conclude my defence that localised bodily sensations involve the bodily field. The argument offered to this effect relies, on the one hand, on the possibility that sensory fields be involved in experiences that do not currently represent external objects – among which, for instance, [Noonday sun]; as well as on the idea that, also in these conditions, sensory fields might be phenomenally salient as such – as it is supposed to happen, incidentally, in [Noonday sun]. I have argued that the phenomenal mark of the sensory field status of the body as experienced in somatosensation is the sense of boundedness.

## Somatosensation and subjectivity

The class of bodily sensations includes states that, phenomenologically, are very diverse: what it is like to proprioceptively feel one’s arm raise is remarkably different from what it is like to feel a sharp pain in the toe, an itch on the thigh, or the touch of a water bottle. Thus, there are reasons to be suspicious about the project promoted here, namely that of subsuming the phenomenology of bodily awareness in all these states to a general notion of bodily field. I will conclude the paper by pointing out what I take to be a central explanatory virtue of this project.

So far, I have developed an argument by comparison: a set of phenomenal features of visual perception typically alluded to as motivation for positing a visual field figure, mutatis mutandis, in a significant subset of bodily sensations. Despite undoubtedly important differences regarding spatial representation accross modalities, there are other features of experiences that sensory fields are posited to capture: how the experiences involve phenomenally relevant properties not assigned to objects, given as properties of an entity that has a special status relative to these objects. I have offered reasons to believe that the body is experienced as this entity in somatosensation.

The theoretical fruitfulness of this comparison goes beyond the matter of how flexible the notion of a sensory field is. As I will suggest in what follows, it touches upon venerable philosophical discussions about bodily awareness in particular.

In the context of visual perception, the idea of properties not being presented as properties of objects intends to capture an intuition about an alleged peculiarly subjective status of some properties involved in visual experience. Take again Slowness in [Bullet train]. In contrast to Rapidity, which is plainly given as a property of the mind-independent train as it moves accross the mind-independent landscape, Slowness concerns the way in which the rapidity of the train at a distance is presented to us qua perceivers. And more than that, Slowness is supposed to *seem to us*, in the experience, somewhat more subjective than Rapidity.

This idea is sometimes explicit in the literature. Siegel (2006, 375) talks about the defence of visual fields as a defence of *mental space*, “home to *apparently* mind-dependent entities” (my emphasis). Brian O’Shaughnessy describes a pure sensation as of the blue sky thus: “something blue, *psychological, and one’s own*” (2000, 468. My emphasis). And Peacocke himself says that “[i]nsofar as we can make sense of a *subjective* space at all, it is precisely such space as is alleged to be involved in the visual field” (2008, 10. My emphasis). In sum, in the notion of a visual field crystalise some features of ordinary visual experiences that, it is supposed, are manifestly subjective, at least under some conditions.

In turn, in recent philosophical literature on bodily awareness we find claims such as the following: “[w]hatever property we can be aware of ‘from the inside’ [somatosensorily] is instantiated in *our own apparent* body … The very idea of *feeling* pain in a limb which *does not seem to be ours* is difficult to frame, perhaps unintelligible” (Dokic, 2003, 325. The first and third emphases are mine); “[t]hanks to their privileged relation to our own body, bodily experiences seem to afford not only awareness of our body *from the inside* but also awareness of our body *as being our own*” (Vignemont, 2018, 12); or “[e]ach of us experiences our own body in a distinctive way. Part of that distinctiveness is that we each experience our body and our limbs *as our own*” (Bermúdez, 2018, 349. My emphasis).

These quotes also articulate an intuition of subjectivity, now relative to somatosensation. Somatosensory perception of the body has been said to involve a *sense of bodily ownership* (Chadha, 2018; Vignemont, 2018; Bradley, 2021; Bermúdez, 2020): bodily properties, as they are perceived in somatosensation, seem to be properties *of me* in a specially compelling way. It seems indeed that, if I perceive a body somatosensorily, I will necessarily self-attribute it.^19^ The discussion is then whether self-attributions of this sort are grounded, strictly speaking, on bodily experiences themselves: whether it makes sense to say that there is something it is like to feel the body as one’s own Martin, 1995; Dokic, 2003; Alsmith, 2015; Peacocke, 2017; Gallagher, 2017; Vignemont, 2018; Chadha 2018; Bradley, 2021). Interestingly, if this were the case – if there is a phenomenology of bodily ownership in somatosensation –, then there is a phenomenal invariance accross somatosensory states despite their phenomenal richness.^20^

My argument in this paper can be read as laying the foundations of an original proposal on the sense of bodily ownership.^21^ The proposal would have it that we experience the body as our own in somatosensation because we experience it as a sensory field. Just as, in the visual domain, the visual field serves as explanans for the peculiarly subjective character of certain properties involved in visual experiences, on this view the bodily field would explain why bodily properties appear somewhat subjective when felt in somatosensation (Brewer, 1995; Dokic, 2003). In both cases, the phenomenology of subjectivity alluded to would consist in the phenomenology of experiencing a sensory field as such.

Developing this account of the sense of bodily ownership, carefully considering its points of contact and divergence with other positions within the debate, is a matter for another paper. It is in order, however, to close this paper by making explicit why the appeal to experiences as of sensory fields arguably constitutes a step forward in our understanding of the phenomenology of ownership.

Prima facie, a sensory field is something intimately linked to *experiences*. As mentioned above, the sensory field of a given experience type structures our access to the objects represented in these experiences (Richardson, 2009). Let us say that sensory fields have an *experience-enabling* role: they are part of the enabling conditions for experiences to represent objects at all. Arguably, the phenomenological difference identified, between the way in which external objects are presented, both in vision and in somatosensation, vis à vis the way in which the sensory field is presented, tracks the distinction between experience-enabling features of experiences, and the objects represented in these experiences. *Experiencing* the visual field is experiencing something that structures our visual access to external objects. Analogously, experiencing the body in somatosensation is experiencing that which eventually structures our somatosensory access to external objects.

This phenomenological feature is unsurprisingly subjective, I contend: experiences are intimately linked to *subjects*. On the one hand, for experiences to occur at all, there need to be subjects that have these experiences. But not only this: on the other hand, in normal conditions, experiences are themselves subjectively marked, at least in the sense that, whenever a subject has them, she will take them to be *her own* experiences – making reports in which she self-ascribes them such as “*I* see a bullet train in the far distance”, “*I* feel the relief of the water bottle”, or “*I* have a headache”. I have just suggested that the phenomenological features of sensory fields identified in this paper track the experience-enabling character of sensory fields. Thus, the suggestion goes, the phenomenology of sensory fields is a phenomenology of subjectivity because it tracks a structuring feature of subjectively marked items, namely *our own* experiences.

In this paper, I have argued that in tactile perception, and in localised bodily sensations more generally, the body is experienced as a sensory field. If this argument is sound, then, plausibly, we own the body in somatosensation inasmuch as we experience it to stand in a peculiar relation to ourselves, by experiencing it as a structuring feature of somatosensation.

## Footnotes

1 It is an essential part of Martin’s view that, in touch and bodily sensation in general, the body is experienced as inscribed within space that goes beyond it, where this space either hosts external objects or is indeed empty. I will elaborate on this aspect of Martin’s view in Sect. 5.2 below. Yet, it is important to note here that Martin writes that, even if “unoccupied regions of space *are relevant* to how one’s body is felt to be”, “they are not themselves *objects of awareness*” (1993, 215. My emphases).

2 For a recent discussion of the significance of the empirical results mentioned in this paragraph for the notion of a tactile field, see Skrzypulec (2022).

3 I will talk indistinctly about *bodily sensations* and *somatosensation*, or *somatosensory states*. By these expressions I refer to the class of phenomenally conscious states that includes pain, proprioception (sensations of bodily posture), kinesthesia (sensations of bodily movement), feelings of bodily temperature, interoceptive sensations (such as hunger or thirst), and sensations related to balance (such as dizziness or vertigo), as well as tactile sensations.

4 For a recent discussion of the epistemic consequences of these differences, see Munro (2021).

5 As an anonymous reviewer has pointed out to me, talk about the body as a sensory field might evoke the way classical phenomenologists have addressed bodily awareness, specially Maurice Merleau-Ponty in *Phenomenology of Perception* (*PP*; 2012) and *The Visible and the Invisible* (*VI*; 1968); and also his predecessor, Edmund Husserl, mostly in *Ideas II* (1989). Indeed, Husserl’s treatment of the body as a “bearer of localised sensations”, which relies on his analysis of touch and its reversibility (specially in ibid., § 36 and ff.), as well as Merleau-Ponty’s own insistence in the reversibility of touch (see e.g. *PP*, 95–96; and *VI*, Ch. 4), resonates with some of the things I will say in Sect. 3 below. Besides, the gist of my defence of the bodily field as emerging from touch, but also from other sensations, might be found in line with Merleau-Ponty’s treatment of corporeality mainly in terms of the body’s perceptual engagement with the world – and more generally with the phenomenological tenet that the body is a perceptual organ (see e.g. *Ideas II*, § 18 and ff.). With this paper I aim at intervening in the debate sketched in this introduction, and I do so in the terms of the philosophers mentioned in the main text. Therefore, a full review of the points of contact between my proposal and those of the phenomenologists, while undoubtedly interesting, is beyond the scope of this paper. For a paper comparing O’Shaughnessy’s and Martin’s analises of corporeality with that of Husserl’s, see Mattens (2009). For a diagnosis of the impact of the phenomenology of bodily awareness for the sense of bodily ownership, both in Merleau-Ponty and Husserl, see Carman (1999).

6 Peacocke calls these properties “sensational properties”.

7 Clark (2000, 53) defines this notion of visual field as “the sum of things *as* represented visually – the ‘intentional content’ of one’s visual representations. To describe this … variety of visual field, describe … what the world would be if it were just as it visually appears to be. This is a treacherous and complicated exercise, but it is often attempted.”

8 By properties *involved in* experiences in this clause I mean, still with Peacocke (2008), properties *relevant to the specification of the experience’s what it is likeness*, of which Slowness and Convergence as discussed in this section are paradigmatic examples. As an anonymous reviewer has pointed out to me, there are properties which arguably meet this Peacockian condition, and which are not intended to be covered by clause (i): for example, the experience starting at some concrete time, its lasting a certain amount of seconds, or its being a particular subject’s experience. Some of these other properties can be filtered out by understanding *experiences* in this definition as equivalence classes of qualitatively identical experiences, which, among other things, can be instantiated by different subjects at different times and places. Still, a full characterization of properties like Slowness and Convergence, which somehow resemble properties attributed to objects, such as Rapidity, yet are not so attributed themselves, is a project that is best left to another paper.

9 However, both on Gibson’s and Peacocke’s accounts, in some cases the visual field might be phenomenally salient as such without the need of an effortful switch of attention. For instance, on their views, the colour properties involved in afterimagery are straighforwardly experienced as properties of the visual field (Gibson, 1950, 31 − 2; Peacocke 2008, 8–9). For more on this issue, see subsection 5.1 below. For critical discussion of this approach to afterimagery, see e.g. Phillips (2013), Gow (2019), and Sarıhan (2020).

10 This definition is instrumental, tailored to the purposes of the argument in this paper: its wording is intended as general enough so that it can be easily transposed from one paradigmatic case of field, namely the visual, to bodily awareness (it refers to the *sensory field*, instead of the *visual* field, and to experience *types*, instead of *vision*). I intend to remain neutral, however, on two fronts. On the one hand, on whether sensory modalities other than the ones discussed here can be said to involve a sensory field. On the other hand, on whether, given sensory fields in other modalities, this definition would be general enough to accommodate them straighforwardly and without qualification. Plausibly, a more encompassing definition would have to be more general, having the definition offered here, and the definitions of sensory fields in other modalities, as specific instances.

11 On Matthen’s view, the proximal and the distal in touch – which he pinpoints terminologically, calling them respectively the *tactile* and the *haptic* – are not merely two aspects of the same experience, but two numerically distinct experiences. Here I intend to remain neutral on this issue.

12 See Skrzypulec (2022) for a related case that bodily representations as they are involved in touch have field-like characteristics.

13 Bermúdez (2020) summarises this in what he calls the *Objective bodily self-presentation thesis*. Other, classical and contemporary expressions of this thesis include Locke ([1689], 1975), Cassam (2011), and Longo (2017).

14 I am thankful to an anonymous reviewer for pressing me on this point.

15 As mentioned above (footnote 9), the approach to afterimages sketched in this paragraph is not uncontroversial. Phillips (2013) argues that afterimages actually do not *appear* independent of objects. On his view, then, the observations about the phenomenology of afterimages brought up in this section on behalf of Peacocke and Gibson are inaccurate. I do not want to commit myself to any specific view of afterimagery in this paper. In fact, I do not need to commit myself to the *phenomenology* of afterimagery being as the visual field defendants claim it to be. What I am interested in, however, are the phenomenological intuitions that defendants of the visual field flag, for, I want to argue, it is cogent to appeal to analogous intuitions in the case of somatosensation.

16 To be clear, nothing hangs on this terminological choice. I find it more natural to talk about the tactile field when dealing with haptic phenomena, and a more general name seems more appropriate when talking about other somatosensory states. As I intend these notions, the tactile field is just a particular case of the bodily field.

17 This is a partial reproduction of Bermúdez’s Boundedness principle. In full, the principle says: “bodily events are experienced within the experienced body (a circumscribed body-shaped volume whose boundaries define the limits of the self)”. For the sake of clarity of exposition, I find it convenient at this point of the paper to omit Bermúdez’s direct reference to the self as part of the principle.

18 For the sake of clarity, the order of the clauses in this section has been slightly changed with respect to the order in Sects. 2 and 3.

19 But see Vignemont (2018, Sect. 2.2 and 2.3) and Bradley (2021) for discussion of the case of somatoparaphrenic patients, who are able to feel sensations in limbs that they claim to disown (Bottini et al., 2002; Moro et al., 2004; Vallar & Ronchi, 2009).

20 At least accross a significant subset of them. Vignemont (2019) argues that interoceptive sensations do not involve a sense of bodily ownership. Interestingly, these sensations are often not localised (Armstrong, 1962).

21 In a similar spirit, Cheng & Haggard (2018) also discuss the contribution that their research on the tactile field makes to the debate on the sense of bodily ownership.
